# Characterization of Product and Potential Mechanism of Cr(VI) Reduction by Anaerobic Activated Sludge in a Sequencing Batch Reactor

**DOI:** 10.1038/s41598-017-01885-z

**Published:** 2017-05-10

**Authors:** Ruofei Jin, Yao Liu, Guangfei Liu, Tian Tian, Sen Qiao, Jiti Zhou

**Affiliations:** Key Laboratory of Industrial Ecology and Environmental Engineering, Ministry of Education, School of Environmental Science and Technology, Dalian University of Technology, Dalian, 116024 China

## Abstract

Bioremediation of Cr(VI) and nitrate is considered as a promising and cost-effective alternative to chemical and physical methods. However, organo-Cr(III) complexes in effluent generally causes environmental concerns due to second-pollution. Here, Cr(VI) reduction and immobilization efficiencies of anaerobic activated sludge were investigated. Anaerobic activated sludge showed strong reduction ability of Cr(VI) and possessed a great potential of Cr(III) immobilization. Almost 100.0 mg l^−1^ Cr(VI) could be completely reduced and immobilized by anaerobic activated sludge in a sequencing batch reactor in 24 h. And most generated Cr(III) was accumulated outside of sludge cells. Extracellular polymeric substances (EPS) could bind to Cr(VI) and form EPS-Cr(VI) interaction to reduce the toxic effect of Cr(VI) and promote the Cr(VI) reduction. Protein-like and humic-like substances were responsible for binding with Cr(VI), meanwhile the process was a thermodynamically favorable binding reaction. Then Cr(VI) was reduced to Cr(III) by membrane-associated chromate reductase of sludge. Eventually, the generated Cr(III) might exist as poly-nuclear Cr(III) complexes adhered to sludge surfaces.

## Introduction

Chromium (Cr) has been widely used in industries including metallurgy, petroleum refining, electroplating, dye making, and manufacture of stainless steel and refractory materials. Improper discharge of wastewater from these industries causes Cr contamination^1^. The essentiality and toxicity of Cr depend on its chemical forms^2^. Cr is most frequently observed in trivalent (Cr(III)) and hexavalent (Cr(VI)) forms in natural water^3^. Cr(VI) (mainly CrO_4_^2−^ at neutral pH or alkaline conditions) has high solubility and bioavailability, and is considered to be mutagenic and carcinogenic, and approximately 100 times more toxic than Cr(III)^2, 4^. Accordingly, almost every regulatory agency worldwide has listed Cr(VI) as a priority toxic chemical^1, 5^. The U.S. Environmental Protection Agency has set the maximum contaminant level for Cr(VI) in drinking water at 100 μg l^−1^. In contrast, Cr(III), the reduced form of Cr(VI), has lower toxicity and is more advantageous to form precipitates at neutral or higher pH^6^. Therefore, reducing Cr(VI) to Cr(III) and removing precipitated Cr(III) by solid separation is a viable mean for Cr(VI) removal from wastewater.

Currently, the co-existence of different oxidized contaminants such as nitrate and chromate is a growing problem and challenge for wastewater treatment. Several methods to remove the co-existing nitrate and chromate have been developed in recent decades, such as chemical reduction, physical-chemical treatments and biological methods^5^. Bioremediation converting these oxidized contaminants to harmless or immobile forms is considered as a promising and cost-effective alternative to chemical process, especially for low-to-mid concentration of Cr(VI) (10–200 mg l^−1^), due to its low cost and ecological compatibility. In a biofilm bioreactor, almost complete removal of nitrate and chromate were achieved^7^. Earlier studies had reported that the simultaneous removal of chromate and nitrate was viable^8, 9^. However, Cr(VI) has toxic effect on microbial activity and more potential to obtain electron donor than nitrate, and the nitrate reduction could be restrained by high Cr(VI) concentration^3, 9, 10^. Compared with biofilm, activated sludge has higher biological activity^7, 11^. Hence, activated sludge could tolerate higher Cr(VI) concentration and achieve simultaneous removal of chromate and nitrate.

Various chromium reduction bacteria have been reported, e.g., *Streptomyces* sp., *Micrococcus* sp., *Pseudomonas sp*., *Bacillus sp*., *Ochrobactrum sp*.^9^. They could apply soluble enzymes and membrane-bound reductase to detoxify Cr(VI) to Cr(III). Additionally, Cr(VI) could also enter cells via the sulphate transport pathway, be reduced by cellular components in the cytoplasm and form reactive species and free radicals that could damage DNA and other biomolecules^9^. Different bacterial strains and reduction environment led to different Cr(VI) reduction mechanism and various Cr(III) location. *Streptomyces* sp. MC1 could reduce Cr(VI) to Cr(III) and accumulate Cr(III) within the cells^12^. Cr(VI) reduction by *Pseudochrobactrum. saccharolyticum* and *Ochrobactrum anthropi* mainly occurred in the extracellular medium^13, 14^. Meanwhile, generated Cr(III) was mostly out of the cells^13, 14^. All components of biofilm could reduce Cr(VI), and most generated Cr(III) was in intercellular space^7^. In a methane-based membrane biofilm reactor, Cr(III) could accumulate inside and outside of bacterial cells, and different Cr(VI)-reducing mechanisms were involved^6^. Therefore, the location of generated Cr(III) is key factor for exploring Cr(VI) reduction mechanism of anaerobic activated sludge. In addition, the characterization of reduction product is also helpful for understanding the reduction mechanism of Cr(VI). The Cr(VI) reduced by *Pseudochrobactrum. saccharolyticum* LY 10 was immobilized in the form of Cr(OH)_3_^13^. Generated Cr(III) by *Ochrobactrum anthropi* formed organo-Cr(III) complexes and had similar coordination with Cr-Gly compound^14^. The location and characterization of generated Cr(III) in anaerobic activated sludge are still unknown, and more investigations are needed.

Earlier researches have focused on exploring the influence of Cr(VI) on the performance and Cr(VI) reducing abilities of microorganisms^15^, but a simple analysis of Cr(VI) elimination rather than direct detection of Cr(III) compound could hardly verify the actual Cr(VI) reducing activity of microorganisms. Cr(III) is more advantageous to form precipitates at pH > 5^9^. In a membrane biofilm reactor, most generated Cr(III) was removed from wastewater in the form of solid Cr(III). However, microbial metabolites could form complexes with Cr(III) and increase the solubility of Cr(III) species^16^. Cr(VI) reduced by *Pseudomonas putida* was partly associated with extracellular polymeric substances (EPS) and remained in the supernatant^17^. More importantly, soluble organo-Cr(III) complexes could be re-oxidized into Cr(VI) in oxidative environments and cause second-pollution^18, 19^. Only considering the removal of Cr(VI) to evaluate the removal efficiency of Cr(VI) is inaccurate, more attentions should be paid to the removal of Cr(III) and total Cr. Currently few studies have been conducted about the Cr speciation in anaerobic activated sludge reactor, therefore Cr speciation after Cr(VI) reduction by anaerobic activated sludge would be explored in the study.

EPS are produced by microbes for a variety of purposes in response to environmental stresses^20–27^. Quantity and composition of EPS have been shown to vary depending upon bacterial strains and environmental stresses^23, 28^. In anaerobic chemostat fed on glucose, exposure to excess Cr(VI) enhanced the EPS production^29^. Similarly, Cr(VI) stimulated the production of microbial EPS by the hydrogen-producing photosynthetic bacteria strain *Rhodopseudomonas acidophila*. Microbial exudates of *Pseudomonas. putida* P18 and *Pseudomonas. aeuroginosa* P16 significantly enhanced microbial Cr(VI) reduction efficiency, and formed soluble organo-Cr(III) complexes to protect the cells and chromate reductase from inactivation^16^. Earlier researchers reported that some negatively charged functional groups in EPS, such as carboxyl and hydroxyl, could effectively bind heavy metal cations and act as a permeability barrier to hinder intracellular penetration of the metal, thus attenuating the toxicity of heavy metals^30–32^. Substantial evidences proved that Cr(VI) would enhance the production of microbial EPS, which enhanced microbial Cr(VI) reduction ability^8, 33, 34^. However, little is known about specific reaction mechanism between Cr(VI) and EPS released by microorganisms. Therefore, this study was conducted to explore the interaction between Cr(VI) and EPS of anaerobic activated sludge in Cr(VI) reduction process.

In this study, an anaerobic reactor was designed to explore the simultaneous removal of nitrate and chromate and influence of different Cr(VI) concentrations on the performance of anaerobic activated sludge reactor. Meanwhile, the distribution of Cr and Cr speciation in anaerobic activated sludge were also taken into account. In addition, special interaction between Cr(VI) and EPS released by microorganisms and the extracellular reducing mechanism of Cr(VI) were also investigated. The results might give a deeper insights into the interaction between Cr(VI) and microbial EPS.

## Results and Discussion

### Performance of the reactor with different Cr(VI) concentrations

**Figure 1:**
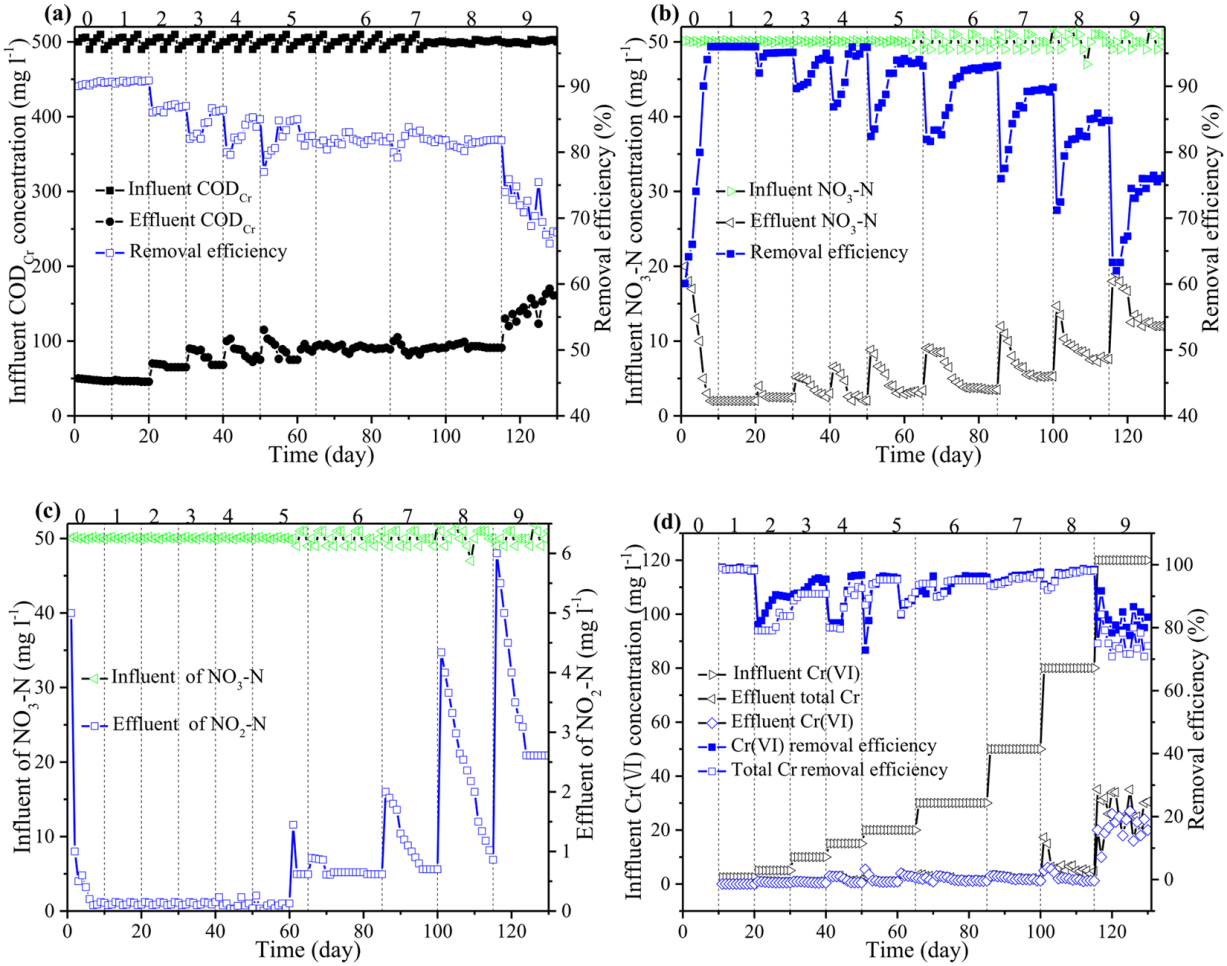
Effect of Cr(VI) on the COD_Cr_, NO_3_-N, NO_2_-N and Cr(VI) removal in the anaerobic reactor. Variations of (**a**) COD_Cr_, (**b**) NO_3_-N, (**c**) NO_2_-N and (**d**) Cr concentrations in the influent and effluent under different Cr(VI) concentrations. The Cr(VI) concentrations were 0.0, 2.5, 5.0, 10.0, 15.0, 20.0, 30.0, 50.0, 80.0 and 120.0 mg l^−1^, corresponding to the phase 0, 1, 2, 3, 4, 5, 6, 7, 8, and 9, respectively.

Anaerobic activated sludge reactor was built and operated for approximately 130 days to explore the effects of Cr(VI) on the performance of the reactor. In the first phase, the reactor was operated devoid of Cr(VI) to acclimatize the synthetic wastewater. Eventually, the reactor showed high and stable removal efficiencies of COD_Cr_ and NO_3_-N, indicating the high activity of activated sludge and strong operating stability of the reactor. The removal efficiencies of COD_Cr_ and NO_3_-N were about 90.5% and 96.0%, respectively. And no accumulation of nitrite was detected. From day 11, Cr(VI) was added into the reactor from 2.5 to 120.0 mg l^−1^. The effects of Cr(VI) on the removal efficiencies of COD_Cr_, NO_3_-N and NO_2_-N in the bioreactor were shown in Fig. 1.

When the concentration of influent Cr(VI) increased from 2.5 to 20.0 mg l^−1^, the removal efficiencies of COD_Cr_ and NO_3_-N showed slight changes. When the concentration of influent Cr(VI) was 2.5 mg l^−1^, the removal efficiencies of COD_Cr_ and NO_3_-N were 90.5% and 96.0%. With the increase of Cr(VI) concentration to 20.0 mg l^−1^, the removal efficiencies of COD_Cr_ and NO_3_-N decreased to 86.6% and 93.1%, respectively. Meanwhile, almost no accumulation of NO_2_-N was observed. Therefore, low concentration of Cr(VI) had no obviously negative effects on the stability of anaerobic sludge and performance of the reactor. When the concentration of influent Cr(VI) was 50.0 mg l^−1^, COD_Cr_ removal efficiency remained at around 78.2%. When Cr(VI) concentration in influent was 30.0 mg l^−1^ and 50.0 mg l^−1^, NO_3_-N removal efficiency decreased in the early stage, gradually increased and remained steady in the later stage. In general, after 20 d of exposure to 30.0 mg l^−1^ Cr(VI), the NO_3_-N removal efficiency increased from 83.3% to 93.1%. After 15 days of exposure to 50.0 mg l^−1^ Cr(VI), the NO_3_-N removal efficiency increased from 73.2% to 89.2%. The increase of Cr(VI) concentration to 80.0 mg l^−1^ resulted in COD_Cr_ removal efficiency slightly increased to 81.5%, which was higher than COD_Cr_ removal efficiency at Cr(VI) concentration of 50.0 mg l^−1^. It might be attributed to the gradual adaption of microbes to the toxic effect of Cr(VI). In addition, needed more electron donors might be needed with the increase of Cr(VI) concentration. Meanwhile, the NO_3_-N removal efficiency had slightly decreased from 89.2% to 85.2%. It suggested that 80.0 mg l^−1^ of Cr (VI) concentration had no significant impact on bioactivity, which was consistent with the finding of Qian *et al*.^11^. In a packed-bed bioreactor, 30.0 mg l^−1^ Cr(VI) led to a decline of the NO_3_-N removal efficiency, and 50.0 mg l^−1^ Cr(VI) concentration caused obvious inhibiting effect on the removal of NO_3_-N^7^. Moreover, biomass activity of pure culture could be depressed by very low Cr (VI) concentration^3^. The discrepancy in these observations could be explained by the fact that different bacterial cultures were used. The inhibition/toxicity of Cr (VI) appears to be more potent in pure cultures than in mixed cultures^15^. Meanwhile, sludge demonstrated higher Cr (VI) reduction capability than biofilm^7, 11^. To further explore the effect of higher Cr (VI) concentration on the performance of reactor and the maximum Cr (VI) reduction capability of sludge, influent Cr (VI) concentration was increased to 120.0 mg l^−1^. Finally, when the concentration of influent Cr(VI) increased to 120.0 mg l^−1^, obvious inhibiting effects on COD_Cr_ and NO_3_-N removal efficiency were observed. COD_Cr_ removal efficiencies plunged to 73.9% and NO_3_-N removal efficiency dropped to 63.3%. After 15 days of operation, the removal efficiency of COD_Cr_ still did not recover and presented a gradually decreasing trend on the contrary. Meanwhile, the removal efficiency of NO_3_-N kept stable around 76.5%. In an expanded granular sludge bed reactor, 120.0 mg l^−1^ Cr(VI) would inhibit denitrification and Cr(VI) reduction capacity of microorganisms and cause NO_2_-N accumulation^8^. Similarly, from day 66, the NO_2_-N concentration of effluent increased obviously. With influent Cr(VI) concentration up to 120.0 mg l^−1^, the NO_2_-N concentration reached the peak value of 6.1 mg l^−1^. Results showed that the activity of activated sludge could be seriously inhibited by high concentration of Cr(VI). In order to evaluate the removal efficiency of Cr(VI) in the reactor, the variations of the influent and effluent Cr(VI) concentration and effluent total Cr concentration were plotted in Fig. 1d. During the whole operation, when the influent Cr(VI) concentration was below 20.0 mg l^−1^, the effluent Cr(VI) and total Cr concentrations were even lower than 1.0 mg l^−1^. Similar trend of the effluent Cr(III) concentration was observed in an aerobic granular sequencing batch reactor^35^. When the influent Cr(VI) concentration was less than 80.0 mg l^−1^, Cr(VI) reduction capacity of the reactor stayed stable with no obvious fluctuation. The increase of Cr(VI) concentration to 80.0 mg l^−1^ led to obvious decline of Cr (VI) removal efficiency to 81.3% and increase of soluble Cr(III) to 2.1 mg l^−1^. Researches showed that the high Cr(VI) concentration of influent would cause inhibition on Cr(VI) reduction and increase Cr(VI) concentration of effluent^7, 8^. After acclimatization, 96.3% of influent Cr (VI) was reduced and 97.7% of generated Cr(III) was removed from wastewater. From Day 116, further increasing Cr(VI) concentration to 120.0 mg l^−1^ led to plunge of Cr (VI) removal efficiency to 74.8% and increase of soluble-Cr(III) to 5.1 mg l^−1^. Eventually, the removal efficiency of Cr(VI) did not increase and remained at about 80.15% with soluble Cr(III) of 2.1 mg l^−1^. Results showed that 120.0 mg l^−1^ Cr(VI) had serious inhibition on NO_3_-N and Cr(VI) reduction. When the Cr(VI) concentration was 80.0 mg l^−1^, simultaneous removal of NO_3_-N and Cr(VI) could be achieved.

In a methane-based membrane biofilm reactor with synthetic mineral medium, almost all of the removed Cr(VI) was converted to Cr(III) solids, meanwhile, effluent Cr(III) concentration was close to the conditional solubility of Cr(III) for pH 7–8^6^. In the whole experiment, the pH of influent was 7.0–7.5, meanwhile the pH of effluent increased to 7.5–8.0 (Fig. S2), which was favorable for Cr(III) to form precipitates. However, effluent Cr(III) concentration was higher than the conditional solubility of Cr(III) for pH 7–8 (i.e., 50–100 μg/L), which might be due to the formation of organic-Cr(III) complexes and increase the solubility of Cr(III). After bio-reduction of Cr(VI), generated Cr(III) could be precipitated or coordinated to active functional sites of bacterial surface/nutrient constituents. In the anaerobic activated sludge reactor, more than 97.2% of generated Cr(III) was almost removed from wastewater, therefore most Cr(III) in reactor might be insoluble. Moreover, the activated sludge in the reactor turned green gradually, and the color of sludge gradually deepened. Meanwhile, no precipitation was observed at the bottom of the reactor. Results showed that solid Cr(III) might be the major product and effectively immobilized by activated sludge.

### Distribution of Cr in activated sludge

**Figure 2:**
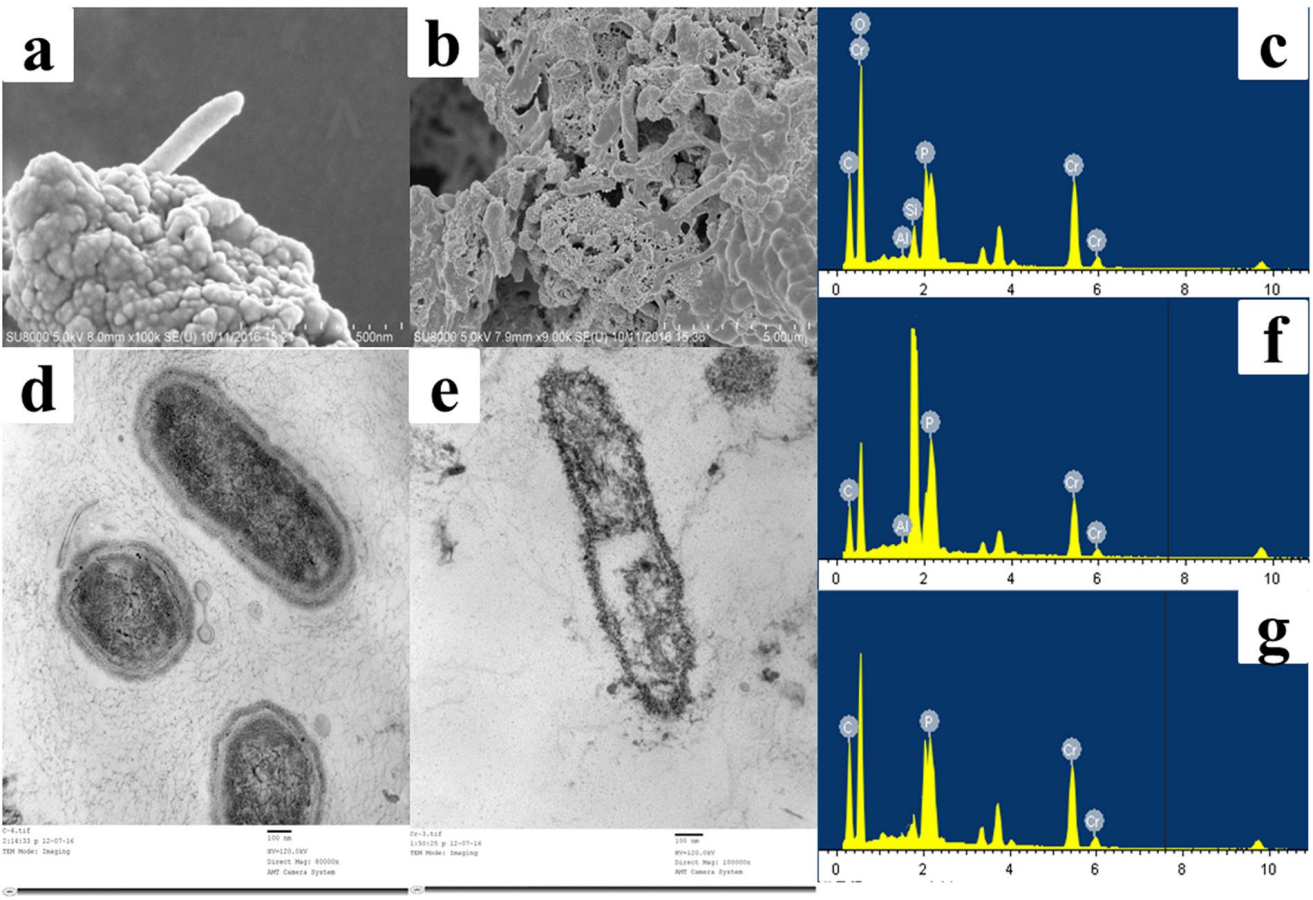
Morphology and element analyses. (**a**) SEM of the activated sludge without Cr(VI); (**b**) SEM of the activated sludge with Cr(VI); (**c**) EDS for the sludge with Cr(VI); (**d**) TEM of the activated sludge without Cr(VI); (**e**) TEM of the activated sludge with Cr(VI); (**f**) and (**g**) EDS for the TEM outside and inside of cell with Cr(VI), respectively. Samples were taken at Day 10 and Day 130.

Scanning electron microscopy (SEM) micrographs of sludge without or with Cr(VI) were presented in Fig. 2. The sludge cells without Cr(VI) treatment (Fig. 2a) were elongated in shape and had smooth surfaces. After incubated with Cr(VI) for 130 days, the sludge cells were coated with granular precipitates (Fig. 2b). The corresponding energy dispersive spectrometer (EDS) (Fig. 2c) with Cr signal suggested that Cr species were available on the cell surfaces. Transmission electron microscopy (TEM) and EDS were applied to analyze the location of the Cr in sludge. Figure 2e showed that cell surfaces were irregular with bulges and wrinkles and precipitates were associated with the cell outer surface. The electron-dense particles represented deposition of generated Cr particles, which were confirmed by Cr signals in the EDS spectra. Meanwhile, strong Cr signal was observed (Fig. 2f) outside the cells and weak Cr signal could be detected (Fig. 2g) inside the cell by EDS analysis, indicating that most Cr were located outside the cells and only very small amounts of Cr were distributed inside the cell. The results were consistent with the SEM-EDS analysis above and implied that membrane-associated chromate reductase might be involved in the Cr(VI) reduction process^36^.

### Proportions of Cr in activated sludge

Proportions of Cr among different compartments of activated sludge were presented in Table 1. As evident, total Cr and Cr(VI) in the control sample were negligible. When 4.32 ± 0.22 mg g^−1^ Cr(VI) was added, almost complete Cr(VI) reduction was achieved. After 15 days of accumulation, about 91.6% of added total Cr was distributed among adsorbed, intercellular and intracellular compartments, and the intercellular represented the major fraction (88.5%). It could be further seen that about 95.4% of total Cr(VI) in intercellular portion was reduced, meanwhile, 80.4% of intracellular total Cr(VI) and 88.9% of adsorbed total Cr(VI) were reduced. With addition of 8.72 ± 0.33 mg g^−1^ Cr(VI), Cr among adsorbed, intercellular and intracellular compartments occupied 90.9% of added total Cr. Approximately 88.5% of remaining total Cr in the activated sludge was in intercellular fraction, and the adsorbed and intracellular fractions only represented about 11.5%. It was also evident that 85.4% of adsorbed and 83.8% of intracellular total Cr(VI) were reduced. When 25.78 ± 3.67 mg g^−1^ Cr(VI) was added, the residue of total Cr in the supernatant was about 15.5%. The residual total Cr was distributed among the sludge compartments. The intercellular total Cr accounted for a large proportion of 94.6%, while the intracellular and adsorbed fractions accounted for just 4.8% and 0.6%. Only 13.6% of adsorbed total Cr(VI) and 21.2% of intracellular total Cr(VI) were unreduced respectively, and 97.9% of intercellular total Cr(VI) was reduced.

**Table 1:** Partitions of Cr(VI) and Cr(III) in the activated sludge compartments after 15 days of incubation. Concentrations of Cr(VI) and Cr(III) are expressed on a dry mass basis of activated sludge. *Represent standard deviation (s.d.).

Total Cr(VI) (mg g^−1^)	Cr(VI) and Cr(III) concentrations in compartments (mg g^−1^)					
	Adsorbed		Intercellular		Intracellular	
	Cr(III)	Cr(VI)	Cr(III)	Cr(VI)	Cr(III)	Cr(VI)
4.32 ± 0.220*	0.0501 ± 0.002	0.0065 ± 0.001	3.356 ± 0.145	0.160 ± 0.030	0.418 ± 0.018	0.127 ± 0.028
8.72 ± 0.330	0.0714 ± 0.002	0.0122 ± 0.003	6.831 ± 0.234	0.175 ± 0.067	0.401 ± 0.038	0.435 ± 0.045
25.78 ± 3.670	0.122 ± 0.039	0.0188 ± 0.004	20.156 ± 3.458	0.435 ± 0.047	0.523 ± 0.063	0.522 ± 0.057

The above data demonstrated that Cr(VI) reduction occurred in all compartments and reduction Cr(III) could accumulate inside and outside of sludge, which were in accord with the results of SEM and TEM. Different Cr(VI) reduction mechanisms were involved the Cr(VI) reduction by anaerobic activated sludge. The intracellular Cr(VI) reduction observed in this study has also been reported by Polti *et al*. using *Streptomyces sp*. MC1^12^. Several studies have reported that most Cr(III) precipitates were located outside the cells and intercellular Cr(VI) reduction was predominant^6, 7^. The intercellular compartment of the activated sludge made the greatest contribution to the reduction of Cr(VI) and immobilization of Cr(III).

### Cr speciation in activated sludge

SEM and TEM results confirmed that most Cr were available on the cell surfaces. However, it was not conclusive that the Cr signal was due to fine granular precipitation of Cr(III). X-ray photoelectron spectroscopy (XPS) was further used to determine the valence state of Cr on the microbes. Two distinct absorption peaks at 588.1 eV and 578.5 eV were observed in Fig. S3, which represented for Cr2p3/2 and Cr2p1/2 hybrid orbital, respectively. The spectra of the sample was consistent with standard Cr(III) spectra, indicating that the Cr adhered to sludge cells was Cr(III). Formation of Cr precipitates has been reported for Cr(VI) reduction in other studies^6, 7^.

The X-ray diffraction (XRD) patterns of sludge with or without Cr(VI) were shown in Fig. S4 in SI. The peaks of sludge in the absence of Cr(VI) might be due to the presence of some polysaccharides and fatty acids. Furthermore, in the XRD pattern of sludge associated with generated Cr(III), no peaks matched with either Cr(OH)_3_ or any other forms of Cr(III) hydroxide. For Cr(VI) reduction by *Bacillus* sp. (CSB-4), the XRD patterns of bacterial cells associated with Cr(III) precipitate also did not match with either Cr(OH)_3_ or any other form of Cr(III) hydroxide^37^. However, this did not rule out the formation of amorphous Cr(OH)_3_ as reduction product, because the color of sludge changed from yellow to green.

Cr(VI) was non-paramagnetic, and did not produce electron paramagnetic resonance (EPR) signal. The EPR signal of Cr complexes was associated with the unpaired electrons of Cr(III). As shown in Fig. S5 in SI, a broad signal centered at a g factor of 1.980 was observed, which could be attributed to the Cr(III) paramagnetic signal in activated sludge. In addition, no other paramagnetic Cr species signal such as that of Cr(V) was found.

Different ligands of Cr(III) complexes resulted in different unpaired electrons environment and changes of EPR signal. EPR signal could be used as a fingerprint to differentiate complexes. Specifically, g-factor of the signal was used to distinguish Cr(III) complexes. Reduction Cr(VI) by *Ochrobactrum anthropic* formed organo-Cr(III) complexes^14^. Organically Cr(III) complexes [Cr-trioxalate, Cr-dioxalate, Cr-citrate, and Cr-EDTA] produced signals in the g = 4–5.5 region^35^. Since the g factor of Cr(III) in sludge was out of region, therefore, generated Cr(III) might not have similar unpaired electrons environment with organically Cr(III) complexes and form organo-Cr(III) complexes^14^. Cr(OH)_3_ had been confirmed as reduction production by *Pseudochrobactrum saccharolyticum* LY10^13^. Meanwhile, inorganic forms of Cr(III)[CrCl_3_ and Cr(OH)_3_] produced broad signals in the g = 2 region of the spectrum^38^. The g factor of standard CrCl_3_ and Cr(OH)_3_ were at g = 1.96^38^. However the g factor of sludge also was not consistent with the g factor of inorganic Cr(III). This was not to preclude the formation of Cr(OH)_3_, because Cr(III) could present as monomeric species Cr(OH)_4_^−^, dimer Cr_2_O_2_(OH)_4_^2−^, trimer Cr_3_O_4_(OH)_4_^3−^ and even tetramer in alkaline media^8, 39, 40^. Gustafsson *et al*. had proved the exist of poly-nuclear Cr(III) complexes (dimer species) in soil^33^. Therefore, generated Cr(III) in reactor might exist in the form of poly-nuclear Cr(III) complexes.

### Reduction mechanism in anaerobic reactor

To confirm the active component for Cr(VI) reduction, the activated sludge, activated sludge without EPS, EPS, and nutrient were introduced into 100.0 mg l^−1^ Cr(VI). The Cr(VI) reducing ability of these components was analyzed respectively. As shown in Table 2, the concentrations of Cr(VI) in activated sludge, activated sludge without EPS decreased quickly. After 24 hour, 98.9% and 70.4% of Cr(VI) were reduced in systems containing activated sludge, activated sludge without EPS, respectively. The Cr(VI) reduction ability of activated sludge without EPS was lower than activated sludge. Meanwhile the Cr(VI) reduction efficiency of activated sludge without EPS decreased gradually. Microbial exudates of *P. putida* P18 and *P. aeuroginosa* P16 had been reported to protect the cells and chromate reductase from inactivation^16^. Results proved that EPS of activated sludge also could protect biomass and reduce the toxic effect on Cr(VI) reduction. Under anaerobic condition, membrane-associated chromate reductase and soluble chromate reductase both could reduce Cr(VI) to Cr(III)^9^. Membrane-associated chromate reductase has been reported in many strains^6^. In EPS and nutrient medium, no significant changes of Cr(VI) concentration were observed, revealing that the EPS and nutrient solution could not reduce Cr(VI) directly. Therefore, it might not be soluble chromate reductase in EPS or nutrient solution but membrane-associated chromate reductase responsible for Cr(VI) reduction by anaerobic activated sludge in this study.

**Table 2:** The Cr(VI) reducing capability of activated sludge, activated sludge without EPS, EPS and nutrient. *Represent standard deviation (s.d.).

Time (h)	Concentrations of Cr(VI) (mg l^−1^)			
	Activated sludge	Activated sludge without EPS	EPS	Nutrient
0	100.05 ± 1.94*	100.12 ± 1.95	100.40 ± 2.35	100.60 ± 2.09
4	72.62 ± 1.29	75.15 ± 2.04	98.97 ± 1.56	99.34 ± 0.98
8	45.53 ± 1.12	55.76 ± 1.57	96.28 ± 1.69	98.24 ± 1.06
12	30.88 ± 1.34	40.25 ± 1.85	94.29 ± 2.12	97.68 ± 1.23
16	10.74 ± 0.89	32.63 ± 1.03	93.47 ± 1.67	97.57 ± 1.78
24	1.05 ± 0.12	30.63 ± 1.47	92.63 ± 2.34	97.55 ± 1.45

To validate whether the reduction process was enzyme-mediated or not, sludge was further treated with heat denaturalization and protein denaturants. As shown in Table 3, the reduction of Cr(VI) could be restrained by heat denaturalization. The phenomenon could be explained that heat treatment destroyed some heat-labile Cr(VI) reductants in the sludge for the disappearance of Cr(VI). To address this alternative explanation, sludge was treated with Hg^2+^, Ag^+^ and surfactants. Furthermore, the data of protein denaturants indicated that both heavy metals (Hg^2+^ or Ag^+^) and surfactants at room temperature significantly inhibited or even ceased the effective reduction of Cr(VI). These results showed that the reduction process was enzyme-mediated and membrane-associated chromate reductase was responsible for the reduction of Cr(VI).

**Table 3:** The Cr(VI) reducing capability of sludge after heating denaturalization and protein denaturant treatments (25 °C). *Represents standard deviation (s.d.).

Time (h)	Concentrations of Cr(VI) (mg l^−1^)					
	Control	5 mM Hg^2+^	10 mM Ag^+^	0.5% SDS	60 °C	90 °C
0	99.50 ± 2.05*	100.78 ± 1.49	100.30 ± 1.60	100.05 ± 2.20	99.60 ± 1.83	99.80 ± 1.83
6	46.65 ± 1.43	98.06 ± 1.94	97.84 ± 2.34	96.26 ± 1.98	75.16 ± 1.60	94.63 ± 2.14
12	6.60 ± 1.33	97.54 ± 2.14	97.05 ± 1.98	95.49 ± 1.08	74.64 ± 1.50	94.09 ± 1.14

### Interaction between Cr and EPS

With the Cr(VI) concentration increased from 0.0 to 50.0 mg l^−1^, the quantity of EPS increased from 231.4 to 1262.9 mg g^−1^ VSS, but it began to decrease at the Cr(VI) concentration of 80.0 mg l^−1^. Finally, at the Cr(VI) concentration of 120.0 mg l^−1^, the quantity of EPS plunged to 459.7 mg g^−1^ VSS and increased by 95.2% than the quantity of EPS without Cr(VI). EPS was mainly composed of protein(PN) and polysaccharide(PS). Under the stress of Cr(VI), sludge would produce more PN than PS. With the concentration of Cr(VI) increased from 0.0 to 50.0 mg l^−1^, PN and PS contents of the sludge increased from 159.0 and 72.3 mg g^−1^ VSS to 723.2 and 418.0 mg g^−1^ VSS, respectively. With up to 80.0 mg l^−1^ Cr(VI), PN and PS of the sludge began to decrease. Eventually, PN and PS gradually decreased to 265.2 and 187.5 mg g^−1^ VSS as the concentration of Cr(VI) increased to 120.0 mg l^−1^. In the whole experiment, the amount of PN had been far more than that of PS.

**Figure 3:**
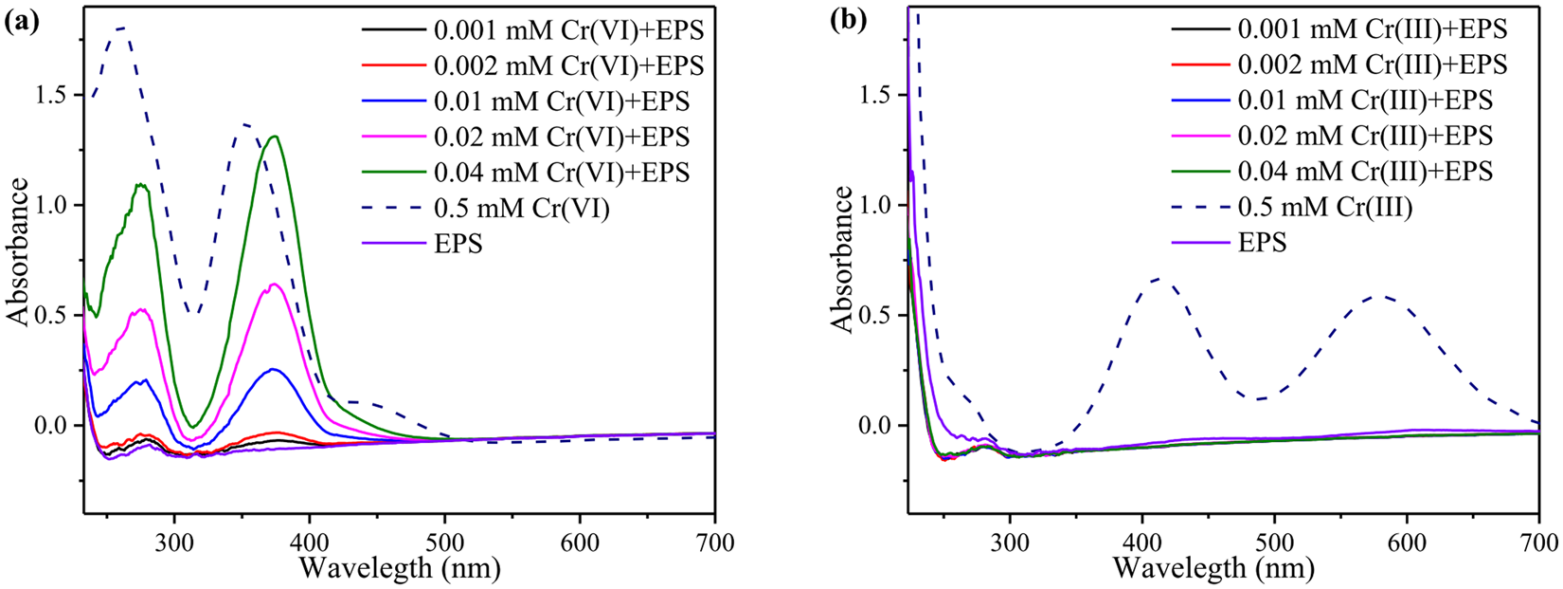
UV-visible spectra analysis. (**a**) Differentiated spectra of EPS, Cr(VI) and the mixture of EPS and Cr(VI) at various Cr(VI) dosages; (**b**) differentiated spectra of EPS, Cr(III) and the mixture of EPS and Cr(III) at various Cr(III) dosages.

To further investigate the interaction between EPS and Cr, the UV-visible absorption spectra of EPS, Cr(VI), Cr(III) and the mixtures were measured. As shown in Fig. 3a, Cr(VI) has two absorption peaks at the wavelength of 257 and 356 nm, respectively. When EPS was added, both peaks showed red shifts (257 vs 273 nm; 356 vs 375 nm) simultaneously. Xu *et al*. also reported that the absorbance spectra of the mixture of EPS and SMZ were different from the sum of EPS and SMZ spectra, indicating the formation of the EPS-SMZ complex^41^. The binding of an auxochrome to a chromophore would cause an increased absorption and a red shift of the chromophore^8, 27^. In the experiment, Cr(VI) acted as the chromophore, and the EPS acted as the auxochrome. As a consequence, the interaction between the EPS and Cr(VI) led to red shifts of Cr(VI) absorption peaks^8^. Wang *et al*. also showed that EPS were able to combine with Cr(VI) and greatly stimulate Cr(VI) reduction due to the protective responses of the bacteria^35^. However, Wang *et al*. did not provide sufficient evidence of the interaction between the EPS and Cr(VI) and explore the main components in EPS responsible for binding with Cr(VI).

For the mixture of EPS and Cr(III), solid precipitation appeared in the solution. Meanwhile, as shown in Fig. 3b, absorption spectra of Cr(III) disappeared, which conceived an explanation that Cr(III) was more likely to precipitate than form EPS-Cr(III) complexes. However, a sharp EPR signal of EPS was observed at g = 4.128 (Fig. S3), which was attributed to organic-Cr(III) complexes^38^. Cr(VI) reduction by *Ochrobactrum anthropic* could also form organic-Cr(III) complexes with organic molecules^14^. However, compared with the Cr(III) signal in sludge, the Cr(III) signal in EPS was negligible, which revealed that organic-Cr(III) complexes with EPS only occupied a small parts.

**Figure 4:**
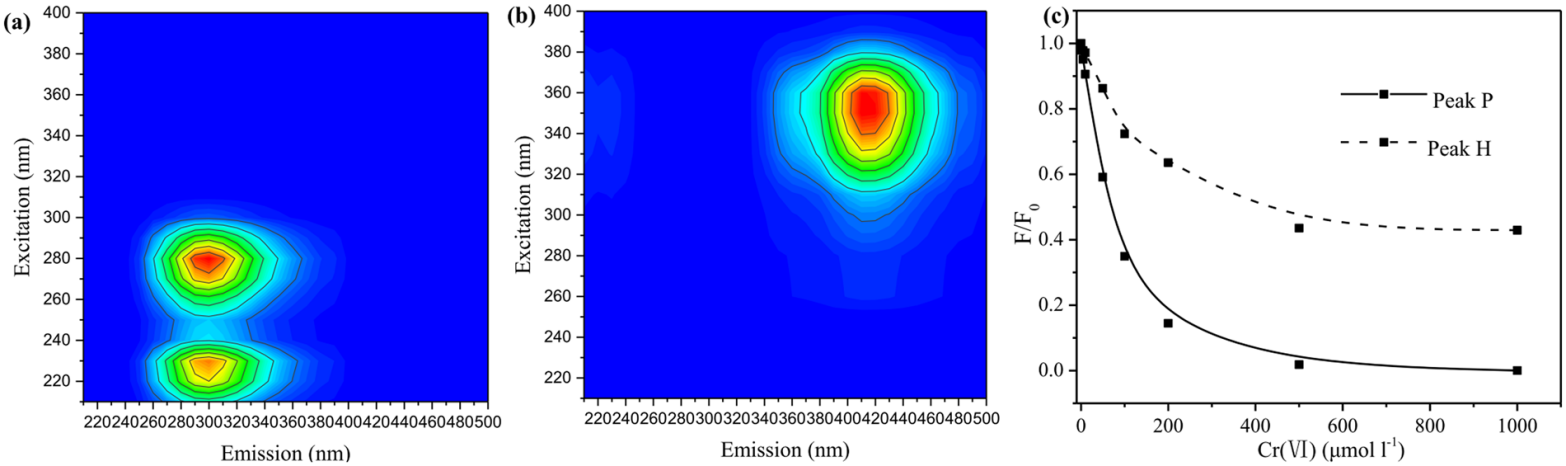
EEM spectra of two main fluorescence components obtained from PARAFAC analysis. (**a**) proteins-like substances; (**b**) humic-like substances; (**c**) their peak intensities of the EEM spectra at various Cr^6+^ dosages obtained from PARAFAC analysis.

Three-dimensional excitation-emission matrix (3D-EEM) fluorescence quenching coupled with parallel factor (PARAFAC) analysis was used to evaluate the interaction between EPS and Cr(VI)^22^. This analysis had been used to evaluate the interaction between EPS and other metal ions (Cu^2+^) or organics (sulfamethazine)^41, 42^. From PARAFAC analysis, two peaks corresponding to proteins-like substances (Peak P, Excitation/Emission 280/310 nm) and humic-like substances (Peak H, Excitation/Emission 350/410 nm) were readily identified from the EEM fluorescence spectra^43^. The addition of Cr(VI) greatly influenced the EEM fluorescence spectra. The PARAFAC analysis further confirmed that the fluorescence peak intensities of two main components in EPS decreased with the increase of Cr(VI) concentration (Fig. 4).

In order to quantify the complexation of Cr(VI) with EPS, Sterne-Volmer equation shown in Eq. 1 was used to calculate the complexation parameters.

1 $$\frac{{F}_{0}}{F}=1+{k}_{q}{\tau }_{0}[Q]=1+{k}_{SV}[Q]$$

*F*_0_ and *F* are the fluorescence intensities of EPS in the absence and presence of Cr; [*Q*] is the Cr concentration, k_q_ is the biomolecular quenching rate constant; τ_0_ is the average lifetime of molecule in the absence of quencher, 10^−8^ s^44^; and k_sv_ is the Sterne-Volmer quenching constant.

As shown in Table 4, the high coefficients of determination implied that this equation could accurately describe the process. From the linear regression of the fluorescence quenching data by PARAFAC analysis, the values of k_sv_ for protein-like and humic-like substances were calculated to be 2.817 × 10^4^ (R^2^ = 0.9594) and 1.25 × 10^3^ (R^2^ = 0.9538) l mol^−1^, respectively. High values of conditional stability constant k_sv_ for the two peaks indicated that EPS was strong ligands to Cr(VI). For EPS and Cr(VI), the k_sv_ value of Peak P was higher than that of Peak H, suggesting the proteins-like substances had a stronger complex ability with Cr(VI) than humic-like substances in EPS. As shown in Fig. 4c, Cr(VI) had more fluorescence quenching effects on proteins-like substances than humic-like substances. Compared with previous studies, the conditional stability constant k_sv_ of Cr(VI) binding to protein-like and humic-like substances in EPS (Table 3) was lower than that of Cu^2+^ ^44^, which might be related to the different characteristics of EPS and metal ions.

**Table 4:** Complexation parameters of Cr with the EPS.

Sample	Cr	Peaks	k_sv_ (×10^4^)	R^2^
EPS	Cr(VI)	P	2.817	0.9594
		H	0.125	0.9538
	Cr(III)	P	0.071	0.9283
		H	0.028	0.9943

In contrast, Cr(III) did not greatly influenced the EEM fluorescence spectra. Cr(III) also formed complexes with EPS with a low conditional stability constant k_sv_ for the two peaks, 0.71 × 10^2^ (R^2^ = 0.9283) and 0.28 × 10^2^ (R^2^ = 0.9943) l mol^−1^, respectively. The k_sv_ values of Cr(VI) were higher than that of Cr(III), indicating that Cr(VI) had a stronger complex ability with EPS than Cr(III).

From the fluorescence quenching data of EPS and Cr(VI) by PARAFAC analysis, the values of k_q_ for protein-like and humic-like substances were calculated to be 2.817 × 10^12^ (R^2^ = 0.9594) and 1.25 × 10^11^ (R^2^ = 0.9538) l mol^−1^ s^−1^, respectively. These values were greater than the maximum diffusion collision quenching rate constant of various quenchers with the biological macromolecules (2.0 × 10^10^ l mol^−1^ s^−1^)^37^. According to previous studies^8, 38^, if the quenching was attributed to collision, i.e., dynamic quenching, the adsorption spectra of EPS and Cr(VI) would not change. Meanwhile, UV-visible absorption spectra showed shifts and revealed the interaction between the EPS and Cr(VI). Therefore, the fluorescence quenching of EPS should be mainly caused by static quenching, specifically, the interaction between EPS and Cr(VI).

**Figure 5:**
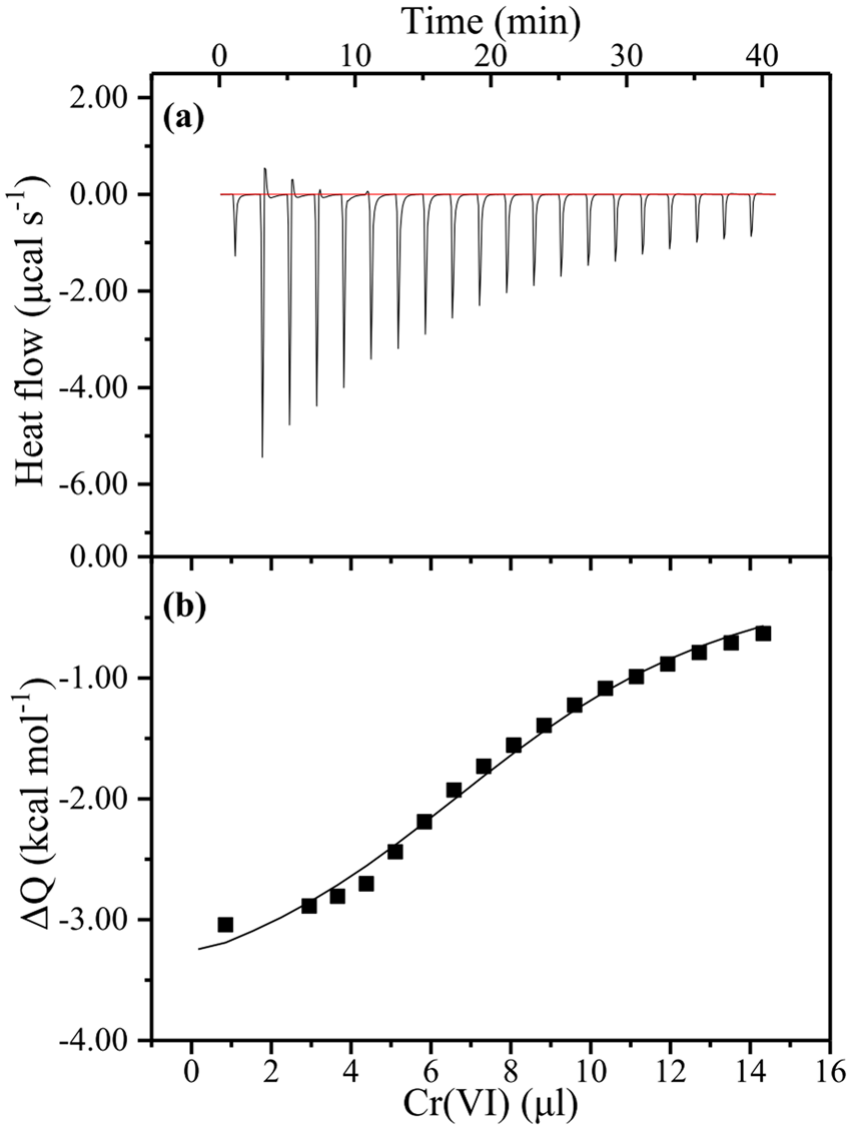
Calorimetric titration of Cr(VI) with EPS in Tris-HCl buffer at 298.15 K. (**a**) Heat released per injection of Cr(VI) in the ITC experiments; (**b**) non-linear regression of the heat vs Cr(VI) dosage.

The reaction between the sludge EPS and Cr(VI) involves two processes in isothermal titration calorimetry (ITC) test: (1) the dilution of Cr(VI) in the solution with a continue dose of Cr(VI); (2) the binding between EPS and Cr(VI). In the experiment, the background calorific effect on the dilution of EPS and Cr(VI) solution was corrected. Thus, the heat released during the experiment was mainly attributed to the binding reaction between the EPS and Cr(VI). Figure 5 showed the profiles of the heat released per injection of Cr(VI), and a thermospositive peak was observed after each injection. From the non-linear regression of the heat vs Cr(VI) dosage, binding constant K, binding enthalpy ΔH between the EPS and Cr(VI) were calculated as 1.27 × 10^4^ l mol^−1^ and −6.25 kJ mol^−1^, respectively.

The binding constant obtained from ITC was a mean value for the binding of Cr(VI) to various compounds in the EPS. The negative value of the binding enthalpy indicated that the binding reaction was an exothermic reaction. The change of Gibbs’ energy of the binding between the EPS and Cr(VI) calculated from the equation 2 was −23.18 kJ mol^−1^, which suggested that the process was a thermodynamically favorable binding reaction.

2 $${\rm{\Delta }}G=-\,RT\,\mathrm{ln}\,K$$

3 $${\rm{\Delta }}H={\rm{\Delta }}G-T{\rm{\Delta }}S$$

From the equation 3, the entropy change of the reaction was calculated as 64.3 J mol^−1^ K^−1^. This result implied that Cr(VI) increased the structure disorder of the EPS. The less value of |ΔH| than |TΔS| inferred that the binding reaction was driven mainly by the entropy change of reaction. Since there were plenty of negatively charged groups on the EPS surface, EPS could readily complex with metals through these groups. This would change the configuration of EPS and decrease fluorescence intensities, as evidenced by both the ITC and EEM results.

Based on these results, Cr(VI) was able to bind with EPS, meanwhile the interaction between EPS and Cr(VI) promoted the disorder of EPS. The protein-like and humic-like substances in the EPS made contributions to the bind with Cr(VI).

**Figure 6:**
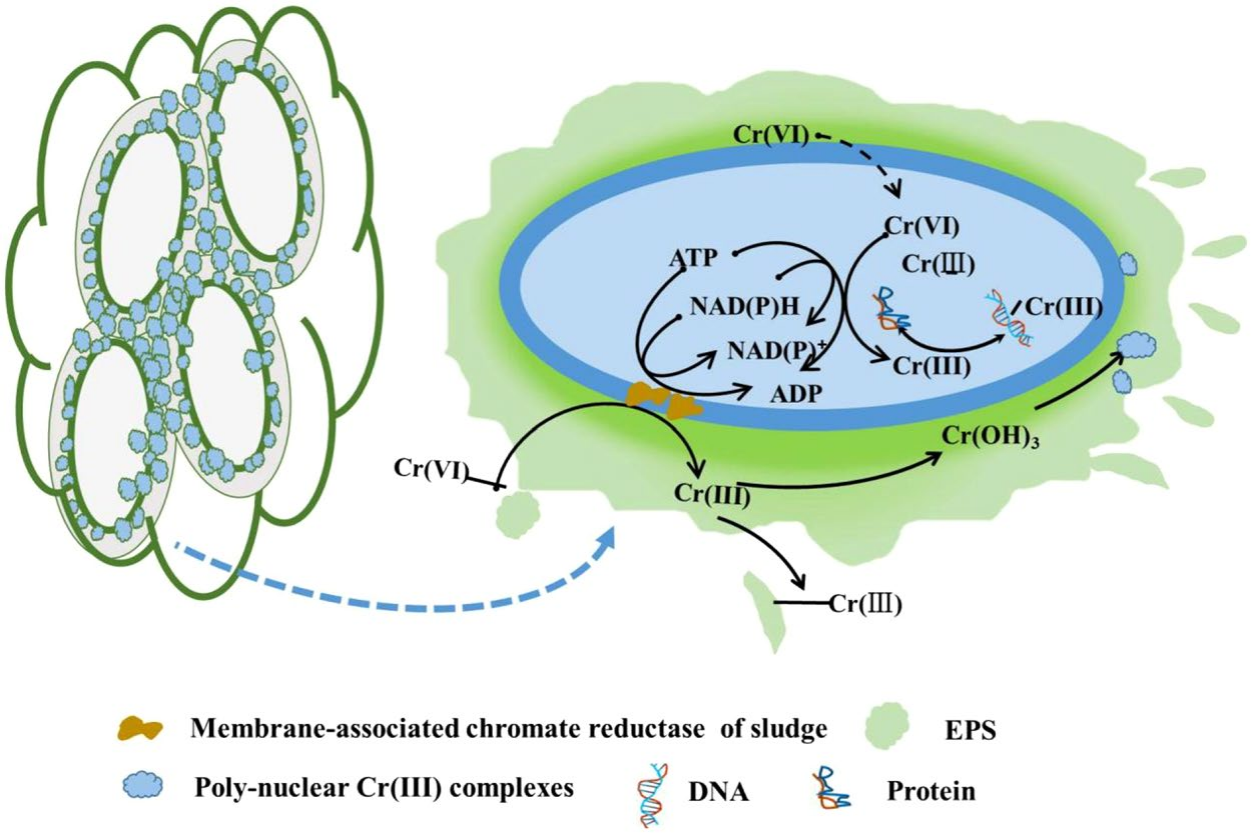
A schematic diagram of Cr(VI) reduction by anaerobic activated sludge.

Activated sludge could reduce Cr(VI) to Cr(III), and membrane-associated chromate reductase of sludge was responsible for extracellular Cr(VI) reduction. Although EPS could not reduce Cr(VI), EPS could bind with Cr(VI) and promote Cr(VI) reduction. Generated Cr(III) in the sludge was amorphous, and most Cr(III) in sludge might exist in the form of poly-nuclear Cr(III) complexes. Based on these analysis, a plausible Cr(VI) extracellular reduction mechanism was proposed in Fig. 6: (1) Cr(VI) was binding to EPS, leading to EPS-Cr(VI) interaction; (2) Cr(VI) was reduced to Cr(III) by membrane-associated chromate reductase of sludge; (3) after bio-reduction, the generated Cr(III) could form poly-nuclear Cr(III) complexes.

## Methods

### Anaerobic reactor experiment and analytical methods

An anaerobic reactor with a working volume of 3 L (inner diameter of 100 mm, height of 500 mm) was constructed to investigate the reduction mechanism of Cr(VI) under a long-term operating cycle. Reactor was fed on the synthetic wastewater with Cr(VI) concentration increased gradually from 0.0 mg l^−1^ to 120.0 mg l^−1^. The composition of synthetic water was shown in SI. In the experiment, glucose acted as carbon source and provided electron for nitrate and Cr(VI) reduction. Nitrate acted as nitrogen source, meanwhile, the simultaneous removal of nitrate and Cr(VI) was also investigated.

The operating cycle was operated automatically with a time-controller for the filling (0.5 h), reacting (22.5 h), settling (0.5 h) and drawing (0.5 h). The influent was pumped into the reactor by the peristaltic pump to keep sludge anoxic. The volumetric exchange ratio of reactor was 0.5. Magnetic mixer was used to ensure that activated sludge mixed evenly. Thermostatic water bath was utilized to keep the temperature at 35 ± 1 °C and promote the growth of microorganisms. In addition, aluminum foil was wrapped outside the reactor to prevent photosynthetic bacterial growth. The reactor schematic diagram was shown in Fig. S1.

Activated sludge was obtained from a local municipal wastewater treatment plant (Chunliu Wastewater Treatment Plant, Dalian, China). Activated sludge was screened through a 2-mm sieve to remove coarse particles and cultivated in the anaerobic reactor. The mixed-liquor suspended solids in the reactor was about 5.26 ± 0.35 g l^−1^.

The effluent was sampled in triplicate every day for the measurement of COD_Cr_, NO_3_-N, NO_2_-N, Cr(VI) and total Cr. The effluent was centrifuged at 10,000 g for (4 °C, 20 min), and supernatant was withdrawn and filtered through 0.22 μm hydrophilic filter membrane for measurement. The concentration of COD_Cr_, NO_3_-N, NO_2_-N and Cr(VI) were measured according to standard method^45^. Total Cr concentration was analyzed by an inductively coupled plasma atomic emission spectroscopy (ICP-AES, Optima2000DV, USA). Soluble Cr(III) concentration could be acquired by deducting the remained Cr(VI) concentration from the total Cr concentration.

### Batch experiments and analytical methods

Four identical 0.5 L reactors (R-A, R-B, R-C and R-D) were constructed to investigate the distribution of Cr in activated sludge. All the experimental conditions were consistent with those of the sequencing reactor experiment. The batch reactors were incubated for 15 days with 0.0 mg l^−1^, 2.5 mg l^−1^, 5.0 mg l^−1^ and 10.0 mg l^−1^ of Cr(VI) for R-A, R-B, R-C, and R-D, respectively. Concentrations of Cr(VI) and Cr(III) were expressed as a dry mass basis of activated sludge, mg g^−1^. And initial concentrations of Cr(VI) were 0.00, 4.32, 8.72 and 25.78 mg g^−1^, respectively. Adsorbed, intracellular and intercellular total Cr concentrations and Cr(VI) concentrations in the activated sludge were determined by the methods in SI^2, 14^.

### Microscopic Characterization of activated sludge

To determine the morphological changes of sludge under Cr(VI) stress and Cr distribution, sludge on Day 10 and Day 130 were collected for SEM and TEM analysis. Sludge samples for SEM were centrifuged at 10,000 g followed by fixation (2.5% glutaraldehyde for 24 h) and dehydration (a graded series of ethanol 30%, 40%, 50%, 60%, 70%, 80%, 90%, 95% and 100%, for 15 min respectively). SEM investigation was performed using a SEM (Nano450, America) coupled with an EDS (51-XMX0013, England) at 200 kV. Sludge samples for TEM analysis were first pre-embedded with 10% calf serum and then pre-fixed by glutaraldehyde, post-fixed by osmic acid, dehydrated with ethanol and embedded in epoxy resins. Then the resins were sliced into ultrathin sections with ultramicrotome (UC7 FCT, America) and next stained the sections with uranyl acetate and lead citrate. Finally, ultrathin sections were observed using a TEM (JEM^−1^ 200EX, Japan) coupled with an EDS (51-XMX0013, England) at 200 kV.

### Characterization of reduction products

To identify the nature of reduction Cr(III), activated sludge on Day 130 was characterized by XPS (PHI 5300, USA), XRD (D/Max 2400, Japan) and EPR (A200–9.5/12, Germany). Activated sludge was centrifuged at 1,500 rpm (4 °C, 10 min) and the pellets were washed twice with 50 mM NaCl for measurement. Then dried sludge samples by air-drying (50 °C overnight) were used for the measurement of XPS and XRD.

XPS was used to analyze the C, O, N and Cr elements. The X-ray source was run at a reduced power of 150 W, and the pressure in the analysis chamber was maintained at less than 108 Torr during each measurement. All of the binding energies were referenced to the neutral C1s peak at 284.6 eV to compensate for surface charging effects. The XPS data were curve-resolved using XPS peak 4.1. The characteristic of reduction Cr(III) was identified by XRD using Cu Kα radiation at a scanning speed of 2° (2θ) min^−1^. The EPR spectra of activated sludge were measured using an EMX spectrometer at 298 K, which was operated at X-band frequency (9.72 GHz) with a 100 kHz modulation frequency. The EPR spectra were recorded at a microwave power of 2 mW and modulation amplitude of 5.0 G.

### EPS extraction

Before extraction, the sludge microorganisms were washed by 50 mM NaCl for three times to remove the residual substrate and metabolic products. The EPS were extracted using the cation exchange resin (CER) the activated sludge samples. After the CER addition, sludge was stirred for 12 h at 200 rpm and 4 °C. Subsequently, the solutions were centrifuged to remove CER and remaining sludge components. The supernatants were then filtered through 0.45 mm cellulose acetate membranes and used as the EPS fraction. The PN and PS fractions in extracted EPS were measured according to the Lowry method with bovine serum albumin as the standard and the anthrone-sulphuric acid method with glucose as the standard, respectively^46, 47^. Concentrations of PN and PS were expressed as per gram volatile suspended solids (mg g^−1^ VSS). EPS for isothermal titration calorimetry test was dried by freeze drying. Then EPS solution of 50.0 mg l^−1^ was prepared before the binding experiments.

### Characterization of active component for Cr(VI) reduction

The activated sludge samples were collected and analyzed when the reactor was operated at a steady state. To confirm the active component for Cr(VI) reduction, the EPS was extracted from sludge when the reactor was operated at a steady state. The extraction of EPS and the preparation of activated sludge, activated sludge without EPS, and EPS were shown in SI. Activated sludge, activated sludge without EPS, EPS and nutrient solution were introduced to 100.0 mg l^−1^ Cr(VI) for evaluating Cr(VI) reducing ability of these components. To confirm whether the reduction process was enzyme-mediated, sludge was treated with heat denaturalization (60 °C/95 °C, 10 min) and protein denaturants, 5 mM Hg^2+^, 10 mM Ag^+^, and 0.5% sodium dodecyl sulfate at room temperature. The treated and untreated sludge were used for Cr(VI) reduction experiments.

### Characterization of interaction between EPS and Cr

The EPS of the sludge samples were extracted and analyzed when the reactor was operated at a steady state. EPS solution of 50.0 mg l^−1^ was prepared before the binding experiments. Then, 0.1 mL of double distilled water, Cr(VI) and Cr(III) solution with various concentrations were added to 10 mL EPS solution and mixed immediately. The pH was adjusted to 7.0 using 0.2 M HCl or 0.2 M NaOH to simulate reactor environment. After binding equilibrium, these solutions were used for UV and EEM spectral analysis to explore the interaction between Cr and EPS.

The UV absorption spectra were recorded in a range of 200–700 nm by a double beam spectrophotometer (UV-4500, Japan) with a 1 cm quartz cell. The 3D-EEM spectra of EPS were measured using a luminescence spectrometry (F-7000, Japan), and the scanning wavelength range was set as: Ex 250–400 nm, Em 300–550 nm. Before analysis, the spectrum of double distilled water was recorded as the blank. PARAFAC analysis was employed to subtract blank, Rayleigh scattering and the overlap of their fluorescence spectra from the samples, separate the actual fluorescence spectra from the EEM fluorescence spectra. The software MatLab7.1 (Math Works Inc, USA) was employed for handling the EEM data.

ITC calorimeter (Microcal iTC 200, USA) was used to investigate binding between EPS and Cr(VI). All solutions were degassed for 15 min under vacuum before titration. Experiments were conducted at 25 °C under stir rate of 300 rpm. The working volume was 240 μL. Titration of Cr(VI) into the EPS solution was performed in 8 μL aliquots injection, with 120s of duration between injections. Analysis of the data was performed by Origin 2016.

## Electronic supplementary material

Characterization of Product and Potential Mechanism of Cr(VI) Reduction by Anaerobic Activated Sludge in a Sequencing Batch Reactor
