# Supplementary material for: Characterization of Product and Potential Mechanism of Cr(VI) Reduction by Anaerobic Activated Sludge in a Sequencing Batch Reactor
Source: Sci Rep. 2017 May 10;7:1681. doi: 10.1038/s41598-017-01885-z (PMC5431812; doi:10.1038/s41598-017-01885-z)
Supplement: Supplementary file 1 — Characterization of Product and Potential Mechanism of Cr(VI) Reduction by Anaerobic Activated Sludge in a Sequencing Batch Reactor [file 41598_2017_1885_MOESM1_ESM.doc]

**Characterization of Product and Potential Mechanism of Cr(VI) Reduction by Anaerobic Activated Sludge in a Sequencing Batch Reactor**

Ruofei Jin, Yao Liu, Guangfei Liu*, Tian Tian, Sen Qiao, Jiti Zhou

Key Laboratory of Industrial Ecology and Environmental Engineering, Ministry of Education, School of Environmental Science and Technology, Dalian University of Technology, Dalian, 116024, China

*corresponding author:

Guangfei Liu, guangfeiliu@dlut.edu.cn


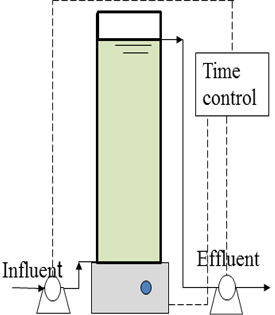


**Fig.** S1**.** Schematic diagram of the anaerobic sludge reactor.

**Fig.** S2**.** pH in influent and effluent of the reactor.

**Fig.** S3. XPS analyses of activated sludge grown with Cr(VI). K_2_Cr_2_O_7_ and CrCl_3_ (both >99% purity) were used as standards for Cr(VI) and Cr(III), respectively. The spectra proﬁles indicate that Cr(III) was the main chromium constituent in the activated sludge.

**Fig.** S4. XRD analysis of activated sludge grown with or without Cr(VI). Activated sludge pellets were washed twice with 50mM NaCl and dried by air-drying (50^o^C, overnight) for the XRD measurement.

**Fig.** S5. EPR analysis of activated sludge with or without Cr(VI) at 298.15K. Activated sludge pellets were washed twice with 50mM NaCl and used for the EPR measurement.

**Fig.** S5. EPR analysis of EPS with or without Cr(VI) at 298.15K. EPS was extracted by CER and used for the EPR measurement.

**Text** S1. Composition of synthetic water

The synthetic water was consisted of glucose 0.936 g NaNO_3_ 0.606 g, Na_2_HPO_4_ 1 g, NaH_2_PO_4_ 1.95 g, MgSO_4_ 0.5 g, NaCl 1 g and trace element solution 2 mL in each liter of tap water. The trace element solution contained the following: Na_2_EDTA 50 g, ZnSO_4_·7H_2_O 2.2 g, CoCl_2_·6H_2_O 1.61 g, FeSO_4_·7H_2_O 5.0 g, Na_2_MoO_4_·4H_2_O 1.1 g, MnCl_2_·4H_2_O 5.06 g, CuSO_4_·5H_2_O 1.57 g and CaCl_2_ 5.5 g in each liter with pH 7.0-7.5. The reagents utilized in the experiments were all analytically pure.

**Text** S2. Determination of adsorbed, intracellular and intercellular total Cr and Cr(VI) concentrations in the activated sludge

After 15 d of incubation, samples were centrifuged for 15 min at 1500 rpm. The supernatant was collected and filtered to determine total Cr and Cr(VI). The activated sludge were quickly washed with 5 mL of 0.05 mol l^-1^ K_2_HPO_4_+0.04 mol l^-1^ NaCl to remove any residue of the synthetic wastewater. Then activated sludge were treated with the mixture (pH 8) of 0.04 mol l^-1^ NaCl and 0.05 mol l^-1^ K_2_HPO_4_ and shaken at 150 rpm for 30 min. After shaking, samples were centrifuged and adsorbed total Cr concentration and Cr(VI) concentration were determined in the supernatant, and adsorbed Cr(III) concentration were calculated through adsorbed total Cr concentration and adsorbed Cr(VI) concentration. While Cr remained in the activated sludge residue was considered as internalized.

For determination of intracellular total Cr concentration and Cr(VI) concentration, liquid nitrogen was added to freeze the desorbed sludge. The frozen sludge samples were ground with a pestle and 10 mL of ultra pure water was added. After filtration of the samples, supernatant was used to determine intracellular total Cr concentration and Cr(VI) concentration. And intracellular Cr(III) concentration was calculated through intracellular total Cr concentration and intracellular Cr(VI) concentration.

To determine intracellular and intercellular Cr(VI) concentration, a known amount of desorbed activated sludge was mixed with 2% NaOH and 3% Na_2_CO_3_ (pH 12) with 1 mol l^-1^ MgCl_2_ added to prevent Cr(III) re-oxidation at 70°C in an ultrasonic bath. After extraction, samples were cooled, ﬁltered and supernatant was used to determine as intracellular and intercellular Cr(VI) concentration. To determine intracellular and intercellular total Cr concentration, dry digestion was applied. A certain amount of sludge centrifuged as above was transferred to a beaker, treated with nitric acid for one night and then placed on the electric heating plate to heat at about 100°C until solid particles were digested. Then, perchlorate was added to eliminate the organics at 200°C until no white smokes were observed. Then the solution treated above was dissolved with 5% hydrochloric acid. Total Cr concentration in the solution was determined as intracellular and intercellular total Cr. As a result, intercellular total Cr concentration and Cr(VI) concentration were calculated from the differences intracellular and intercellular total Cr concentration and Cr(VI) concentration and intracellular total Cr concentration and Cr(VI) concentration. And subsequently, intercellular Cr(III) concentration were calculated through intercellular total Cr concentration and intercellular Cr(VI) concentration.

**Text** S3. Preparations of activated sludge, activated sludge without EPS, and EPS

Before experiments, the sludge microorganisms with Cr(VI) were washed by 50mM NaCl for three times to remove the residual substrate and metabolic products. The sludge samples were centrifuged at 10,000 rpm (4^o^C) for 10 min, then two identical wet sludge samples were used for the experiment. EPS was extracted from one sludge sample as mention above. Then, the residual sludge was used as sludge without EPS. The other sample was as activated sludge with EPS.
